# Dark Chocolate Intake Acutely Enhances Neutrophil Count in Peripheral Venous Blood

**Published:** 2017-07-01

**Authors:** Martina Montagnana, Elisa Danese, Gabriel Lima-Oliveira, Gian Luca Salvagno, Giuseppe Lippi

**Affiliations:** 1 *Section of Clinical Biochemistry, University of Verona, Verona, Italy*

**Keywords:** Chocolate, Cocoa, Leukocytes, Neutrophils, Infection

## Abstract

Beside the well-established impact on decreasing the risk of cardiovascular diseases (1), recent attention has been paid to the relationship between cocoa-containing foods and the immune system (2), showing that dark chocolate consumption enhances the systemic defense against bacterial (3) and viral (4) infections. Hence, the current study aimed at investigating the acute effect of dark chocolate intake on peripheral blood leukocytes.

**Dear Editor**

All participants provided an informed consent for participating in this study, which was carried out in accordance with the Declaration of Helsinki, and approved by the local ethics committee. The study population consisted of 18 consecutive healthy male volunteers (37±10 years), recruited from the laboratory staff, in alphabetical order. Exclusion criteria were history of hematological disorders, abnormalities of routine blood tests and signs or symptoms of disease, including weakness, cough, and fever. Each volunteer ingested 50 g of 90% cocoa chocolate (Noir Prodigieux, Lindt, Kilchberg, Switzerland) within 3 to 5 minutes. The nutrition information of the 50 g chocolate was as follows: 1242 kJ (i.e., 296 kcal), 27.5 g of lipids (15 g of saturated fat acids), 7 g of carbohydrates (3.5 g of sugars), and 5 g of proteins. Blood was drawn, immediately before chocolate intake and 4 hours after consumption, in K_2_EDTA evacuated blood tubes (Vacutest Kima, Padova, Italy). All subjects accomplished 8 hours of overnight fasting before chocolate ingestion, and were then refrained from eating, drinking, and smoking for the next 4 hours after chocolate intake. White Blood Cell (WBC) count and differential count was performed using Siemens ADVIA 2120 (Siemens Healthcare Diagnostics, Tarrytown NY, USA). Differences of laboratory data before and after chocolate intake were evaluated with paired Wilcoxon’s signed-rank test and Bland-Altman plots (Analyze-it Software Ltd, Leeds, UK).

The results of this study are shown in Table 1. 

A significant increase of both WBC and neutrophils counts was observed 4 hours after dark chocolate intake, whereas the other parameters of WBC remained unmodified. The Bland-Altman plot analysis revealed a mean increase of 17% (95% CI, 12% to 23%) for WBC and 14% (95% CI, 6% to 22%) for neutrophils, respectively. 

**Table 1:** White Blood Cell Count and Differential Count Four Hours after Dark Chocolate Ingestion in Eighteen Ostensibly Healthy Subjects

Parameter	Baseline	Four hours after dark chocolate ingestion	
	Mean±SD	Mean±SD	p
White blood cells (x10^9^/L)	5.9±0.9	7.0±0.9	<0.001
Neutrophils (x10^9^/L)	3.4±0.8	3.9±0.7	0.001
Lymphocytes (x10^9^/L)	2.1±0.7	2.3±0.5	0.056
Monocytes (x10^9^/L)	0.4±0.2	0.4±0.1	0.379
Eosinophils (x10^9^/L)	0.2±0.2	0.2±0.2	0.146
Basophils (x10^9^/L)	0.1±0.1	0.1±0.1	0.086
LUC (x10^9^/L)	0.1±0.1	0.1±0.1	0.234

SD, standard deviation; LUC, Large and Unstained Cells

In conclusion, the acute effect of dark chocolate intake should be regarded as a potential source of biological (preanalytical) variability (5). Furthermore, a recent study showed that acute cocoa consumption decreases the expression of adhesion molecules (6), thus leading to reduced migration of neutrophils in peripheral tissues (including atherosclerotic plaques), which would hence contribute to reduce local inflammation, whereas their increase in the circulation may promote more efficient response against pathogens.
